# Subcutaneous Heparin Leads to Rectus Sheath Hematoma: A Rare Complication

**DOI:** 10.7759/cureus.2769

**Published:** 2018-06-08

**Authors:** Muhammad Azharuddin, Mridul Gupta, Mihir Maniar

**Affiliations:** 1 Internal Medicine, Monmouth Medical Center, Long Branch, USA

**Keywords:** subcutaneous heparin, hematoma, rectus sheath, anticoagulation, vte, anemia, dual antiplatelet therapy, bleeding

## Abstract

Rectus sheath hematoma (RSH) is a rare complication that usually occurs in patients receiving anticoagulation therapy. It can mimic an acute abdomen and be life-threatening. RSH can develop even with prophylactic dose of heparin. Early recognition is necessary to decrease morbidity and mortality. RSH should be considered in anticoagulated patients who develop sudden onset of abdominal pain. RSH is usually managed conservatively, but sometimes requires surgery. Patients who are taking antiplatelet require careful monitoring with the use of anticoagulation (AC). It is important to identify them early. This is a case of 69-year-old female who presented with epigastric pain secondary to rectus sheath hematoma. She was receiving subcutaneous injections of heparin for left lower quadrant pain and swelling for venous thromboembolism prophylaxis. Ultrasound of abdomen revealed large rectus sheath hematoma.

## Introduction

Rectus sheath hematoma (RSH) is a rare complication that usually occurs in patients receiving anticoagulation (AC) therapy. It can mimic an acute abdomen and be life-threatening [1-2]. Major bleeding complications includ­e abdominal wall hematoma, intrahepatic hemorrhage, psoas or thigh hematoma, retroperitoneal hematoma, and spinal or epidural hematoma [3].

RSH was reported upon treatment with intravenous UH and vitamin K antagonists. However, RSH is rarely linked with the use of subcutaneous heparin or enoxaparin [3]. Recently, providing prophylactic low molecular weight heparin (LMWH) and unfractionated subcutaneous heparin has increased for hospitalized patients and is suggested for deep vein thrombosis (DVT) prophylaxis. Therapeutic dosing is viewed as the standard medical care for many prevalent diagnoses including pulmonary embolism, acute DVT, acute coronary syndrome, and atrial fibrillation [4].

RSH is reported to have overall mortality of 4% [3]. Many cases were reported of RSH secondary to LMWH. However, very few cases are reported of RSH due to low dose unfractionated heparin. RSH is usually managed conservatively, but sometimes requires surgery. It is important to identify them early [2]. This is a case of a patient with RSH due to subcutaneous heparin.

## Case presentation

A 69-year-old African American female presented with complaints of epigastric pain and 10-pound unintentional weight loss over three months. Abdominal pain was dull, non-radiating, localized in epigastric region and was not associated with food intake. She also had associated occasional non-bloody, non-bilious vomiting and early satiety. Three weeks ago, upper gastrointestinal (GI) endoscopy showed mild antral gastritis for which she was started on proton pump inhibitor (PPI). Her past medical history was significant for chronic kidney disease stage IV (baseline creatinine 1.6–2 mg/dl), anemia of chronic disease, chronic obstructive pulmonary disease (COPD), two ischemic strokes, right internal capsule infarct three years ago, left lacunar infarct six months prior with no significant residual neurological deficits, and esophageal carcinoma treated 14 years prior with chemotherapy and radiotherapy. She also used to consume hard liquor on daily basis for most of her life and smoked three packs per day for 40 years. There was no family history of any bleeding disorders. Her medications prior to this admission included aspirin, clopidogrel, omeprazole, amlodipine and atorvastatin.

**Figure 1:**
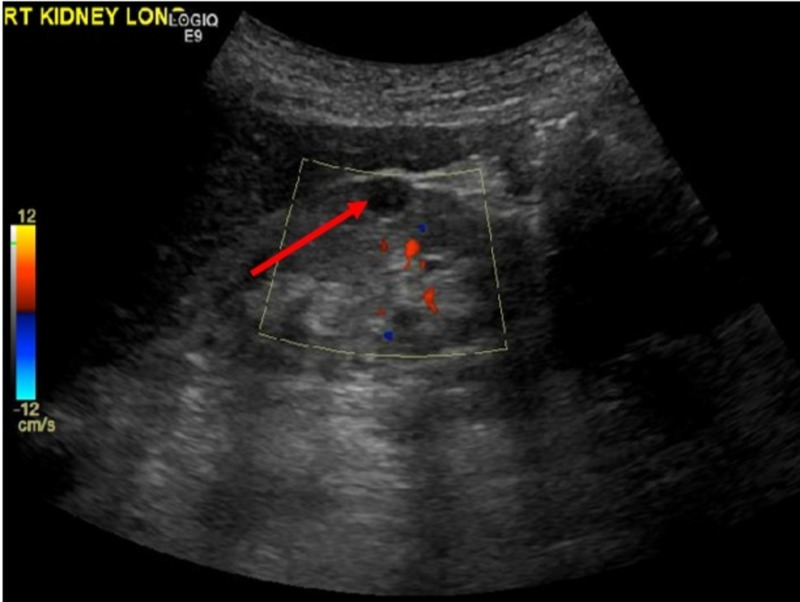
Ultrasound of right kidney. Arrow indicates 0.8 x 0.9 x 0.9 cm cystic lesion in mid-pole region.

**Figure 2:**
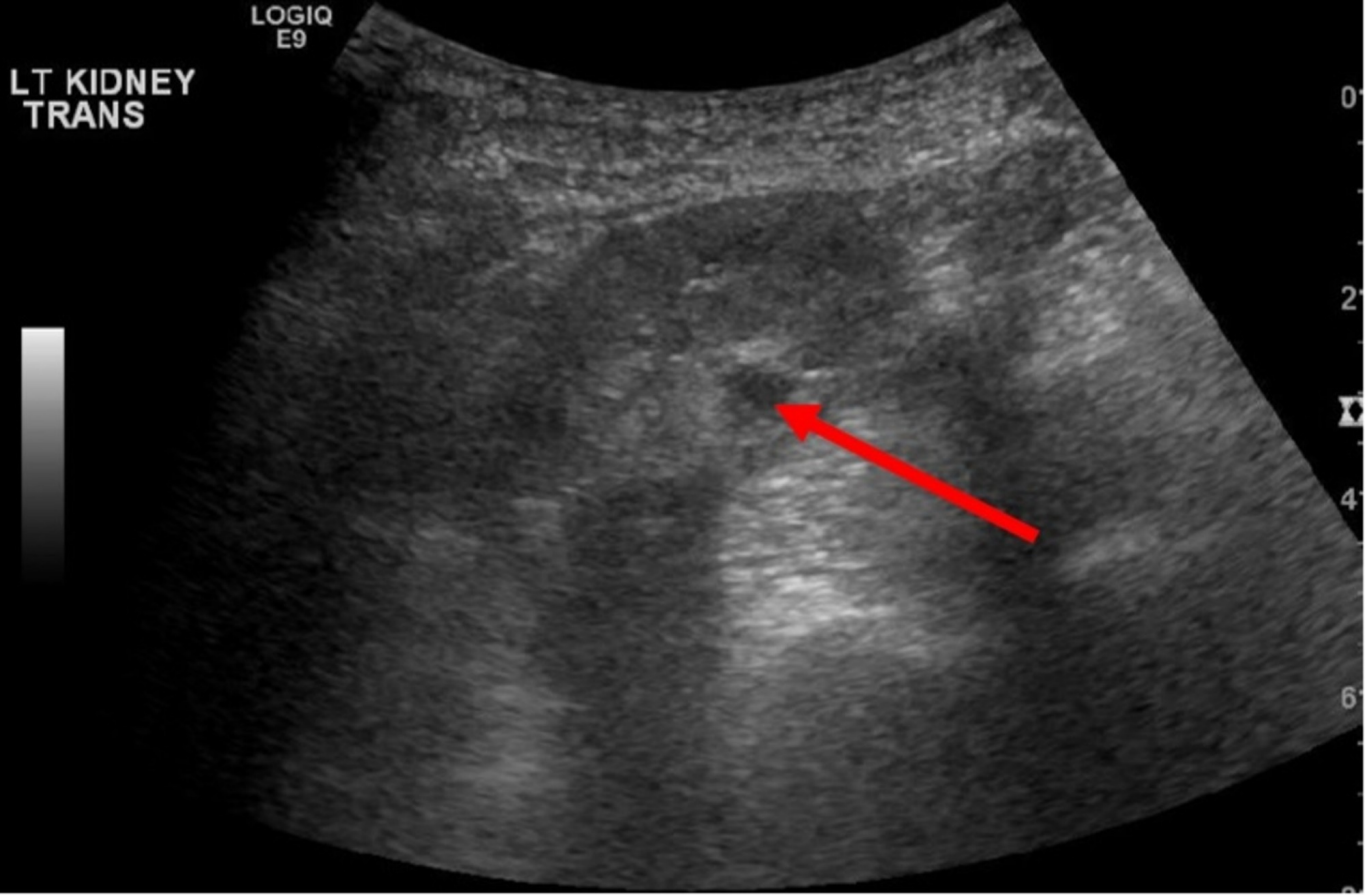
Ultrasound of left kidney. Arrow indicates 1.2 x 0.9 x 1.0 cm cyst in mid-pole region.

On presentation, the patient was hemodynamically stable. Abdomen was soft with some epigastric tenderness, but without any palpable masses. Neurological examination was non-focal. Hematological investigations from admission was suggestive for hemoglobin 8.2 g/dl, total leukocytes 6300 cells/mm^3^, peripheral blood eosinophilia (12%), normal anion gap metabolic acidosis with serum bicarbonate 13 mEq/L and potassium 4.1 mg/dl, serum lipase 94 IU/L, albumin 5.0 g/dl, total protein 8.9 g/dl. Iron studies obtained for evaluation of anemia showed normal iron level and iron saturation with ferritin elevated to 738 ng/ml. Urinalysis showed 30 mg/dl protein, pH 5.5, small blood, too-numerous-to-count white cells, 0 to 5 red cells per high-power field. Creatinine at admission was elevated at 6.94 mg/dl with blood urea nitrogen (BUN) 83 mg/dl. Her bloodwork done three months prior to admission showed hemoglobin of 10.1 g/dl. At that time, renal ultrasound had demonstrated both kidneys to be atrophic with bilateral renal cysts. The kidneys were echogenic and atrophic. There was a 0.8 x 0.9 x 0.9 cm cystic lesion in mid-pole of the right kidney (Figure 1). There was a 1.2 x 0.9 x 1.0 cm cyst noted in the mid-pole of the left kidney (Figure 2).

The patient was admitted for acute kidney injury and was started on intravenous fluids with bicarbonate. Intravenous ceftriaxone was given for possible urinary tract infection. Urine was also found positive for presence of eosinophils raising concern for acute interstitial nephritis (AIN) from PPI, for which omeprazole was changed to famotidine. Computed tomography (CT) scan of chest, abdomen and pelvis was pursued to rule out any malignancy but was limited study in absence of intravenous contrast. Underlying renal dysfunction limited the use of contrast. It confirmed the presence of previously known emphysematous changes in lungs and diverticulosis coli. No omental or abdominal masses were demonstrated. With conservative management, creatinine improved to about 5.1 mg/dl and plateaued.

On fourth day of admission, the patient developed new left lower quadrant pain and swelling. She was receiving subcutaneous injections of heparin 5000 IU every 8 hours at that site for venous thromboembolism (VTE) prophylaxis. She has received a total of 10 doses of heparin prior to this event.

**Figure 3:**
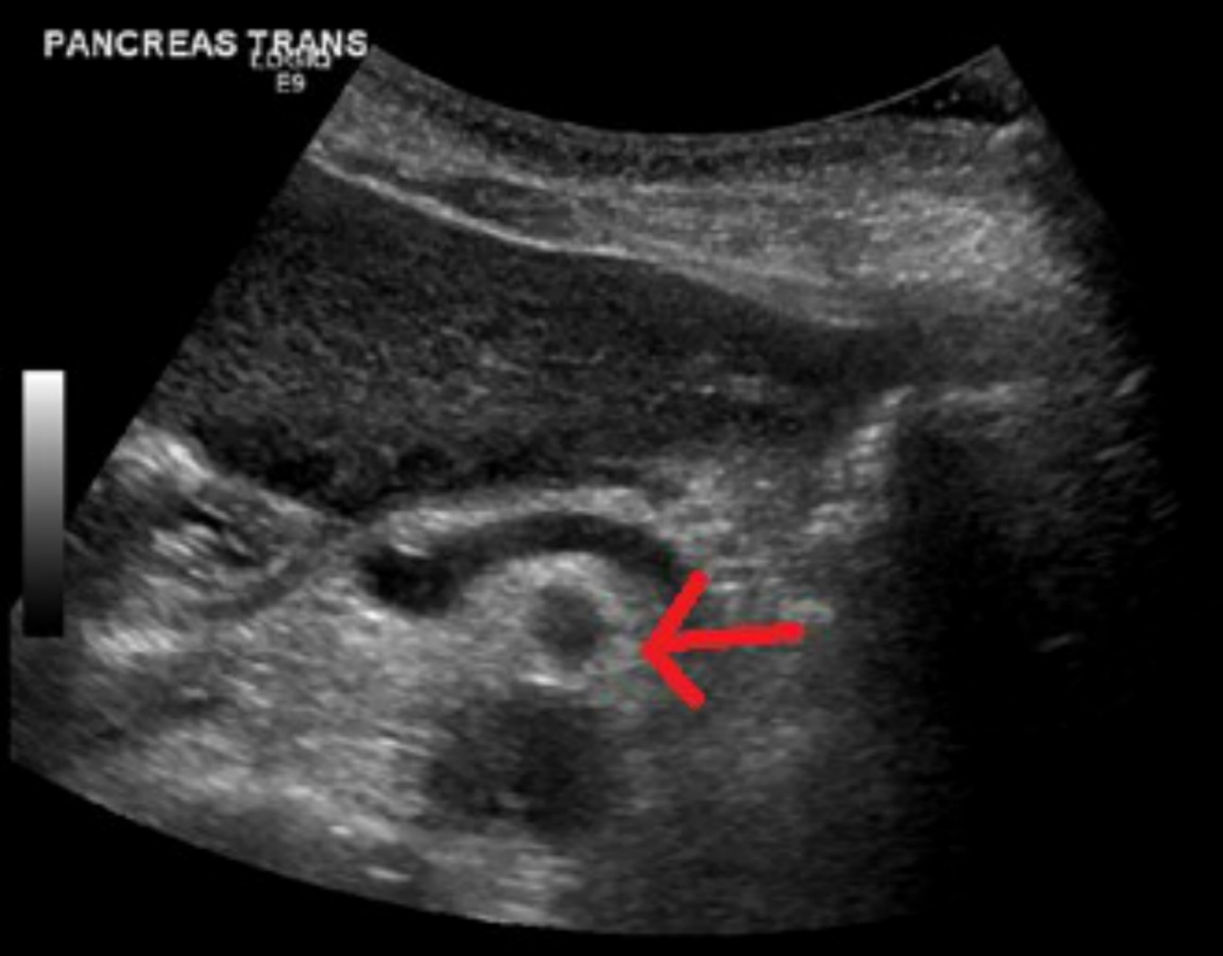
Ultrasound of abdomen; heterogenous hypoechoic mass in subcutaneous tissues of left lower quadrant.

Subcutaneous hematoma was suspected and aspirin, clopidogrel, and heparin products were stopped. Ultrasound of abdomen identified heterogenous hypoechoic mass measuring 10.2 x 6.4 x 4.8 cm (craniocaudad x width x depth) in subcutaneous tissues of the left lower quadrant, extending inferiorly from the periumbilical area concerning for large rectus sheath hematoma (Figure 3).

Her hemoglobin dropped down to 6.6 g/dl requiring 3 unit packed red cell transfusion. Her platelet count never dropped down and coagulation studies were normal.

Oral prednisone was also started at 40 mg daily for possible AIN from PPI use, and was continued for four weeks. Patient’s renal function did not improve with conservative management for which the decision was made to start her on renal replacement therapy. Right internal jugular vein Perm-A-cath was placed to initiate hemodialysis. Clopidogrel was resumed after one week and aspirin was resumed after two weeks of developing rectus sheath hematoma.

Patient’s hemoglobin at discharge was 10.3 g/dl and remained stable on follow-up two months later. Her renal function did not improve despite corticosteroid therapy, and she was eventually transitioned to peritoneal dialysis six months later.

## Discussion

RSH is an uncommon but serious bleeding complication linked with anticoagulant and antiplatelet treatment (APT) [1,3]. It is a rare entity caused by blood accumulation within rectus abdominis muscle (RAM)’s sheath [3].

Symptoms of RSH include abdominal pain that increases with movement, nausea, and vomiting. Differential diagnosis includes diverticulitis, appendicitis, cholecystitis, incarcerated inguinal hernia, ovarian cyst torsion, or acute pancreatitis. Physical exam usually displays a painful, firm abdominal mass consistent with the rectus sheath. Ecchymosis typically does not appear until two to five days after the hematoma [4-5]. In 70% of cases, RSH occurs in the lower abdomen [3].

The pathogenesis of RSH involves the anatomy of the anterior abdominal wall. The RAM is wrapped anteriorly and posteriorly by strong aponeurotic sheaths of the external and internal obliques, and transversus abdominis muscles above the arcuate line that is in between the umbilicus and the symphysis pubis. Since the epigastric vessels lie between the RAM and posterior rectus sheath, most RSHs above the arcuate line occur posterior the RAM [6].

Risk factors of RSH include AC, blunt trauma, muscular exertion, older age, thin body habitus, central obesity, pregnancy, female, recent abdominal surgery, external trauma, medical conditions causing coagulopathy, persons receiving several types of abdominal subcutaneous injections, specific comorbid diseases, use of con­current medications, and renal insufficiency [3-4,7]. Using AC and APT in patients with atrial fibrillation increases major bleeding risk. There is a high number of patients with chronic kidney disease (CKD) who had RSH [7].

Some reports suggest RSH is a result of impaired coagulation from administering subcutaneous lose dose of heparin or as a direct result of involuntary deep administration of heparin. The direct mechanism may be of inadvertent intramuscular injection or epigastric vessel disruption [2].

Ultrasonography is the preferred initial imaging, as it is cost-effective, has a sensitivity of 70% for RSH, and displays collection within abdominal wall. CT scan is considered the gold standard diagnostic modality, as it has sensitivity of up to 100%, and can detect, localize, and quantify the extent of the abdominal wall hematoma and other intraperitoneal structures [2-3,7].

In hemodynamically stable patients, conservative management is suggested, including discontinuation of heparin, analgesia, correction of the anticoagulation state, volume replacement, and red blood cells transfusions. For unstable patients, an aggressive treatment with angiographic selective embolization is necessary. If this is unsuccessful, bleeding vessel ligation may be performed [8]. Surgical management should be reserved to eliminate strangely large RSHs or for patients with embo­lization failure mainly due to increased infection risk and impaired breathing or mobility [3].

AC or APT should be discontinued until the active bleeding is controlled. Platelet transfusion can benefit any bleeding patients taking antiplatelet agents after the agents have been suspended for a certain time [7].

## Conclusions

In conclusion, RSH is an uncommon complication of subcutaneously administered low dose unfractionated heparin. Very few cases have been reported so far. The presentation is characteristic, and clinicians should be suspicious and make prompt diagnosis.
